# Stem cell aging in adult progeria

**DOI:** 10.1186/s13619-015-0021-z

**Published:** 2015-10-03

**Authors:** Hoi-Hung Cheung, Duanqing Pei, Wai-Yee Chan

**Affiliations:** 1 CUHK-CAS GIBH Joint Research Laboratory on Stem Cell and Regenerative Medicine, School of Biomedical Sciences, Faculty of Medicine, The Chinese University of Hong Kong, Shatin, Hong Kong S.A.R., China; 2 Chinese Academy of Sciences (CAS) Guangzhou Institutes of Biomedicine and Health (GIBH), Guangzhou, China; 3 The Chinese University of Hong Kong, Room G03A, Lo Kwee-Seong Intergrated Biomedical Science Building, Shatin, N.T., Hong Kong S.A.R., China

**Keywords:** Werner syndrome, Stem cells, Aging, WRN

## Abstract

Aging is considered an irreversible biological process and also a major risk factor for a spectrum of geriatric diseases. Advanced age-related decline in physiological functions, such as neurodegeneration, development of cardiovascular disease, endocrine and metabolic dysfunction, and neoplastic transformation, has become the focus in aging research. Natural aging is not regarded as a programmed process. However, accelerated aging due to inherited genetic defects in patients of progeria is programmed and resembles many aspects of natural aging. Among several premature aging syndromes, Werner syndrome (WS) and Hutchinson–Gilford progeria syndrome (HGPS) are two broadly investigated diseases. In this review, we discuss how stem cell aging in WS helps us understand the biology of aging. We also discuss briefly how the altered epigenetic landscape in aged cells can be reversed to a “juvenile” state. Lastly, we explore the potential application of the latest genomic editing technique for stem cell-based therapy and regenerative medicine in the context of aging.

## Werner syndrome: clinical features, genetics, and pathogenesis

Werner syndrome (WS) was first described by Otto Werner in 1904. Patients of WS are characterized by premature aging which are clinically apparent during 20–30 years old. WS patients show abnormal physical development such as a bird-like face, short stature, and slender limbs. They also display a high-pitched voice, remarkable loss and graying of hair, and scleroderma-like skin changes. Other common clinical presentations include bilateral cataracts, type 2 diabetes mellitus, hypogonadism (with reduced fertility), skin ulcers, premature arteriosclerosis, osteoporosis, and cancer predisposition [1, 2]. Ninety percent of WS is linked to mutations on *WRN*, a member of the *RecQ* family responsible for stable genome maintenance. Owing to autosomal recessive inheritance, biallelic mutation on *WRN* is pathogenic. The frequency of WS is estimated to be 1 in 20,000–40,000 in Japanese population and slightly lower in the world [3, 4].

The pathogenesis of WS due to loss of the WRN protein has been well elucidated by the biochemical nature of the WRN helicase. As a multifunctional nuclear protein, WRN is an ATP-dependent 3′–>5′ helicase and exonuclease. It unwinds secondary DNA structure such as tetraplex DNA and Holliday junction and resolves stalled replication fork during DNA replication. More importantly, WRN participates in multiple DNA repair pathways such as base excision repair, non-homologous end joining, and homologous recombination [5]. In addition to DNA replication and DNA repair, WRN is also involved in telomere maintenance. Telomere replication and protection are pivotal for maintaining genome integrity and stability and also serve as an aging marker. Accelerated aging due to loss of WRN function is well explained by its biochemical functions in relation to DNA replication, repair, recombination, and telomere maintenance [6]. From a developmental point of view, progressive cell loss due to apoptosis, cell cycle arrest, or senescence in actively dividing cells may be a consequence of WRN loss. Since WS is an adult onset disease, genetic instability accumulates with age. The manifestation of premature aging phenotypes becomes apparent when accumulated DNA damages are not properly repaired and WRN-deficient cells fail to maintain their genomic integrity [7]. WS cells thus, while being diagnosed and biopsied, display a variegated translocation mosaicism in skin fibroblasts and shorter telomere length [8]. WS fibroblasts also display premature senescence and accelerated telomere loss. From the view of pathogenesis, accumulation of deleterious DNA mutations and persistence of genomic instability eventually attain a pathogenic threshold to be reflected in different phenotypes - premature aging in many of the mesenchymal cell types and acquisition of neoplasm [9].

## Stem cell aging in connection with segmental progeria in Werner syndrome

Progeroid syndromes such as WS and Hutchinson–Gilford progeria syndrome (HGPS) show phenotypes of accelerated aging resembling normal aging, such as the development of bilateral cataract, aging skin, graying and loss of hair, cardiovascular disease, and osteoporosis [1]. However, they are segmental in nature, meaning that only a specific category of tissues is predominantly affected. For WS, age-related dementia and cognitive impairment are rarely reported, leading to the hypothesis that progeroid syndromes are not seemingly an accelerated mode of aging. Nevertheless, how de novo mutation in, for instance, *WRN*, leads to segmental aging remains to be answered. A number of models of aging have been described to explain the aging mechanisms in stem cells as well as in model animals. These models are based on the molecular pathways known to regulate longevity, reactive oxygen species (ROS) production, mitochondrial function, telomere protection, cell cycle control and senescence, protein homeostasis, and systemic inflammation [10]. In WS, specific pathways are amplified and connected to the aging program leading to accelerated but lineage-specific aging. Our discussions below focus on the telomere function and epigenetic regulation in connection with stem cell aging in progeroid syndromes.

## Telomere dysfunction as a hallmark of premature aging in WS cells

The hypothesis of telomere erosion as a chronological aging marker has been well documented by many investigations linking the telomere lengths to the biological age. A population in advanced age has a shorter average telomere length than younger groups, although variations exist within each age group [11]. In telomerase-null mice, shorter telomeres are found after several generations of interbreeding. Degenerated phenotypes are observed in late-generation, telomerase-null mice. However, reconstitution of telomerase can rescue these phenotypes, suggesting the telomere length as a pathological factor for aging [12]. Telomere is not only a biological clock counting the number of cell divisions but also a protective structure for ensuring genomic integrity and stability. Specifically, the telomere is highly organized and orchestrated by telomere-binding proteins called shelterin [13]. Loss of telomeric DNA or failure of telomere protection elicits telomere dysfunction, which subsequently induces cell cycle arrest, apoptosis, or senescence [14]. Telomere dysfunction-induced cell cycle arrest in stem cells serves as a protective mechanism to prevent propagation of genetically unstable daughter cells from passing on [15]. In many adult stem cells such as mesenchymal stem cells (MSC) and muscle stem cells, telomerase activity is restricted, indicating a finite number of divisions allowed. The adult stem cell pool is kept at a balanced quiescent and proliferative state in response to signals of differentiation or regeneration. The stem cell pool is believed to exhaust with age, thus giving less regenerative potential [16]. In laboratory mice, the longest telomeres are found in stem cell compartments requiring active proliferation such as hair follicle and small intestine stem cells [16].

How the WRN-deficient progeroid stem cells become depleted remains a challenging question to answer, mainly because adult stem cells from WS are difficult to acquire. A comparison of the in vivo telomere length from a cohort of WS patients with normal individuals demonstrates accelerated telomere attrition in muscles and skins [17]. Depletion of WRN protein in normal fibroblasts also shows a similar result, leading to the hypothesis that telomere dysfunction due to WRN deficiency is the primary cause for WS pathophysiology [18]. The mouse model for WS with *Wrn* deletion, however, is not sufficient to recapitulate the classical features of WS in human [19]. Such species-specific difference can be ascribed to the fact that laboratory mice possess a longer telomere reserve than human. In support of this notion, *Wrn* knockout mice in the background of critically short telomeres (G4-G6 *Terc* ^*−/−*^ *Wrn* ^*−/−*^), but not in mice with normal telomeres (*Terc* ^*+/−*^ *Wrn* ^*−/−*^), display aging phenotypes reminiscent of human WS [20]. However, WRN may not be an essential factor for telomere replication because telomerase-immortalized WS cells can extend the telomere length without inducing telomere dysfunction and senescence [21].

## The role of WRN in telomere maintenance

The WRN protein plays a diverse role in protecting genome stability through interacting with different proteins in several pathways (refer to review by Bohr et al.) [5].

The current view suggests that WRN interacts with shelterin proteins to resolve G-quadruplex and Holliday junction structures in addition to aiding repair of DNA breaks during DNA synthesis [6, 7, 22]. G-quadruplex structures are common when DNA polymerase is synthesizing the G-rich, single-stranded D-loop found at telomeres. Consistent with this notion, WRN-depleted cells show defective synthesis at the lagging strand of sister telomeres perhaps due to the failure to resolve these structures at telomeres [23]. Eventually, accelerated and progressive telomere loss is found in WS cells, particularly in adult stem cells which are required for continuously repairing damaged tissues throughout life. Using redifferentiated stem cells derived from WS induced pluripotent stem cells (iPSCs), telomere dysfunction and sister telomere loss at the lagging strand are also observed in MSC [24]. Interestingly, redifferentiated neural progenitor cells are not affected, suggesting a lineage-specific aging mechanism in relation to the telomere function. Many questions remain open as to how different adult stem cells in WS, like hematopoietic stem cell (HSC), hair follicle and dermal stem cells, muscle and cardiovascular stem cells, and even germ cells, are selectively affected by WRN loss. One possible explanation for the segmental aging mechanism in progeroid syndrome is the differential telomerase activity in various adult stem cells. Telomerase activity is detected in germ cells and adult stem cells such as neural stem cells and HSC but almost absent in MSC. Telomerase is highly expressed in embryonic stem cells and iPSC as well as in many cancers [25]. In support of this, iPSCs reprogrammed from WS fibroblasts have normal telomere length that prevent cells from entering senescence. Differentiated cells, including MSC and fibroblasts, however, become senescent prematurely whereas they can be rescued by ectopic expression of telomerase [24, 26]. Therefore, telomerase may be a factor counteracting the telomere loss/dysfunction in the absence of the WRN helicase.

## Aging-associated epigenetic alterations in normal aging and progeroid syndromes

**Fig. 1:**
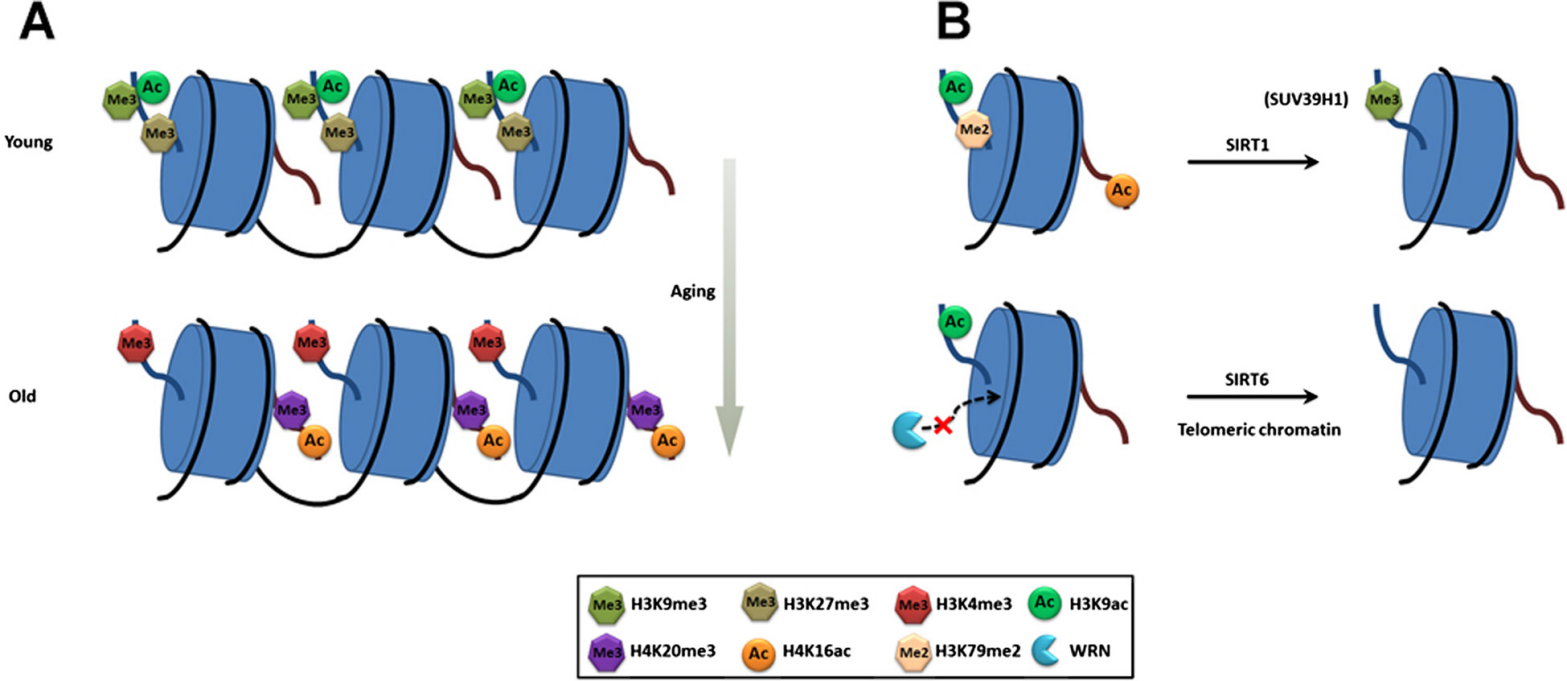
Aging-associated epigenetic changes on histone modifications. **a** In aged somatic and stem cells, chromatin is progressively changed. H3K4me3, H4K20me3, and H4K16ac are increased whereas H3K9me3, H3K27me3, and H3K9ac are decreased. Chromatin remodeling proteins (e.g., HP1α and NuRD) and DNA methylation are also decreased globally (not shown). Changes of chromatin structure and organization affect transcriptional activity and genomic stability related to aging. **b** SIRT1 and SIRT6 are important aging regulators. SIRT1 deacetylates H3K9 and H4K16 and increases H3K9me3 through SUV39H1. SIRT6 also deacetylates H3K9 at telomeric regions. Hyperacetylation of telomeric H3K9 impairs association of the WRN protein with telomeres, hence, leading to premature aging

Epigenetic modifications on DNA and histones change with age and vary in different cell types, disease states (e.g., cancers), and developmental and differentiating stages [27]. Epigenetic marks that link to aging include gain of H3K4 and H4K20 trimethylation, H4K16 acetylation, and decrease in H3K9 and H3K27 trimethylation (Fig. 1a) [28]. H4K20 trimethylation is a marker for constitutive heterochromatin. Increase of H4K20 trimethylation is found in aging tissues of rats and also in patients of HGPS [29, 30]. Epigenetic changes on histone in WS are largely unknown. A recent report suggests that heterochromatin alteration, as reflected by global loss of H3K9 trimethylation and reduction of SUV39H1 (protein that trimethylates H3K9) and HP1α, is found in *WRN* knockout cells [31]. The group concludes heterochromatin disorganization is a potential determinant of premature aging in WRN-deficient cells.

DNA methylation is also drifted in aged cells. Globally, hypomethylation is found at various organs/cell types with advanced age, for examples, blood and dermal fibroblasts. Repetitive sequences such as *Alu* and *LINE-1* show decreased 5mC content with age, suggesting a mechanistic link to the increased genomic instability due to the loss of global methylation [32]. However, some locus-specific regions, especially for those at CpG islands, show hypermethylation as cells age [33]. Some of the hypermethylated genes are putative tumor suppressor genes, extrapolating that epigenetic silencing is another risk factor for increased neoplastic events in elderly people. By profiling a number of WS and HGPS patients, aberrant DNA methylation profile is detected. For WS, differential methylation on CpG sites is located in genes enriched for the IKB kinase/NF-kappaB signaling and proteinaceous extracellular matrix formation [34]. These candidate genes may be involved in the phenotypic changes observed during premature aging. Interestingly, *WRN* itself is also controlled by epigenetic regulation. Epigenetic downregulation of the *WRN* gene is found in many age-related diseases, e.g., cataract and cancer [35, 36].

Unlike genetic mutations, epigenetic modifications are reversible, raising the question of whether aging and longevity can be changed if we are able to change the age-associated epigenome. A family of genes known to promote longevity are actually enzymes for epigenetic modifications. The sirtuin gene family plays a critical role in regulating aging. Among the seven mammalian sirtuin genes, *SIRT1*, *SIRT3*, and *SIRT6* have been shown to improve health through regulating diverse processes [37]. Here we briefly discuss the roles of *SIRT1* and *SIRT6* in stem cells and aging.

As a histone deacetylase, SIRT1 not only deacetylates H4K16 and H3K9 but also regulates the histone methyl-transferase SUV39H1 during heterochromatin formation [38]. Loss of SIRT1 reduces H3K9 trimethylation and impairs localization of heterochromatin protein 1 (HP1), an epigenetic alteration associated with aging. Although *Sirt1* overexpression in mice does not increase longevity, it does improve healthy aging such as wound healing and reduced incidence of cancer [39]. In another study, *Sirt1* overexpression specifically in the brain extends lifespan through upregulation of *Ox2r* by cooperation with Nkx2-1 [40]. In addition to the epigenetic regulatory role, Sirt1 also participates in repairing DSB in response to oxidative stress and helps to combat genomic instability and age-dependent transcriptional changes [41]. The role of SIRT1 in regulation of adult stem cell aging and homeostasis is evident. *SIRT1*-deleted young HSCs show skewed differentiation with reduced lymphoid compartment, anemia, and expression pattern similar to aged HSCs [42]. In human MSC derived from various adult tissues, SIRT1 demonstrates a beneficial effect on long-term growth and differentiation potential [43, 44]. Because SIRT1 is able to delay premature senescence, it also plays a role in progeria. Lamin A, the mutant form causing laminopathy-based premature aging, interacts with and activates *Sirt1*. Such interaction is interrupted in progeroid cells, leading to prominent decline of adult stem cells in the progeria mouse model [45]. For WS, expression and localization of WRN is modulated by SIRT1 and PML [46]. SIRT1 is reported to deacetylate WRN [47]. Acetylation of the WRN protein enhances its stability by inhibiting ubiquitination [48]. SIRT1 affects translocation of the WRN protein from nucleolus to nucleoplasm when DNA repair is required [49].

*SIRT6*, another important member of the sirtuin family, has other unique biological functions. SIRT6 expression declines significantly in the aged brain, which is associated with increased H3K9 acetylation [50]. Decreased SIRT6 expression is also found in HGPS and senescent cells but unknown for WS cells [51]. *Sirt6* knockout mice exhibit premature aging and developmental abnormalities including profound lymphopenia and metabolic defects [52]. On the other hand, overexpression of Sirt6 increases lifespan in male but not female mice. Transgenic male mice have a lower serum level of IGF1, higher level of IGF-binding protein 1, and altered phosphorylation levels of major components of IGF1 signaling [53]. Restoring SIRT6 expression in HGPS cells impedes premature senescence and formation of dysmorphic nuclei [51]. SIRT6 is a histone H3K9 deacetylase that maintains repressive telomeric chromatin. Repressive heterochromatin at telomeres is an epigenetic mechanism for silencing telomere-proximal genes, which is disrupted in the absence of SIRT6 [54]. Mechanically, SIRT6 specifically associates with telomeres where it deacetylates H3K9 which is required for stable association with WRN. SIRT6-depleted cells show telomere dysfunction with premature cellular senescence that resembles WS cells [55]. Besides the role in telomere function, SIRT6 also helps maintain genomic stability and regulates metabolic homeostasis, another two hallmarks of aging [56]. In summary, *SIRT1* and *SIRT6* are the most well-known *Sir2* orthologs that contribute to aging partially through epigenetic regulation of the “aging” epigenome (Fig. 1b).

## Reprogramming aging epigenome for regenerative medicine

The epigenetic makeup not only becomes changed with aging but also defines cell identity during development and stem cell differentiation. Research from the last decade underscores the reversibility and plasticity of changing cell fates through epigenetic reprogramming [28]. This is achieved through resetting the epigenome by transferring a differentiated somatic nucleus to enucleated egg using somatic cell nuclear transfer technique or by expressing pluripotency-associated transcription factors (OCT4, SOX2, KLF4, and c-MYC or variant combinations) [57]. Insights gained from the reprogramming experiments raise the feasibility of erasing epigenetic memories associated with aging to restore a “juvenile” state. The epigenome of iPSC, in fact, is highly similar to embryonic stem cells (ESC) and capable of generating a variety of adult stem cells with regenerative capacity [58]. Epigenetic reprogramming is achieved initially using embryonic somatic cells [59]. Subsequently, somatic cells from more aged donors can be reprogrammed as well [60]. Moreover, skin fibroblasts from progeroid syndromes including HGPS and WS can be successfully reprogrammed to iPSC [24, 26, 61, 62]. One of the exciting findings from these reprogramming experiments is the erasure of aging phenotype originally associated with premature aging in the progeroid cells, despite the presence of the disease genotypes in those cells. Interestingly, premature aging phenotypes, such as expression of progerin proteins in HGPS and telomere dysfunction in WS, reappear when reprogrammed iPSCs are differentiated to somatic and adult stem cells. These iPSC-based disease models, although limited to in vitro condition, are useful for studying the aging mechanism in different adult stem cell compartments.

Epigenetic reprogramming to iPSC is accompanied with reactivation of telomerase activity, which is generally silenced in somatic cells [63]. Telomerase reactivation is usually found in cancers and ESC. These immortal cells require telomerase to replenish eroded telomeres to support active proliferation. Therefore, the acquisition of telomerase activity during reprogramming can be regarded as a process of “rejuvenation”. Intriguingly, iPSCs not only reactivate their telomerase activity but also acquire telomeric epigenetic marks characteristic of ESC. This is demonstrated by the decreased H3K9 and H4K20 trimethylation and increased telomere recombination as compared to differentiated MEF [64]. We also found the restoration of telomere function during reprogramming in WS cells. This is accomplished by two events, the reactivation of telomerase genes and the expression of shelterin genes [24]. Telomerase activation is beneficial to WRN-deficient cells as it elongates short telomeres and prevents accelerated senescence [65]. Secondly, proper expression of shelterin genes ensures telomere capping and prevention of telomere dysfunction. In line with this notion, reprogrammed WS cells do not exhibit premature telomere loss or defective synthesis of sister telomeres at their lagging strands (these are the two important features of WS cells) nor exhibiting premature senescence [24, 26]. In HGPS cells expressing progerin, reprogramming also silences progerin expression in HGPS iPSC lines, although it is unknown whether it is related to telomerase [61].

Telomere reprogramming appears an attractive tool for reversing aging, including progeria cells [63]. However, senescence and other aging phenotypes (e.g., genomic instability, abnormal nuclear structure, defective DNA repair, and accelerated telomere loss) recur as the pluripotent cells are differentiated to adult stem cells, limiting the use of these stem cells for regenerative purpose [61]. Genomic editing may provide a solution for this purpose. Recent advancement in technologies such as TALEN and CRISPR/Cas9 facilitates precise genetic engineering for generating mutant or correcting mutant cells. A number of patient-specific iPSC lines have been successfully corrected in different disease models to restore the normal phenotypes [66]. Currently, monogenic diseases serve a good model for genetic correction, although the issues on safety and ethics have to be refined [67]. The majority of WS is monogenic with mutations found in the *WRN* locus [68]. Although genetic correction of *WRN* has not been reported in WS cells, the availability of WS iPSC makes this approach possible [24, 26]. Indeed, restoration of wild-type WRN expression would rescue the aging-associated phenotypes caused by WRN deficiency, as demonstrated by multiple studies that lentiviral/retroviral delivery WRN gene can rescue the phenotypes [18, 69, 70]. Genetic correction of *WRN* may not have a significant beneficial effect on iPSC and its pluripotency, since the high telomerase activity in iPSC appears to “fix” the telomere-associated problems [24]. However, restoration of wild-type WRN is beneficial to adult stem cells or differentiated somatic cells because telomerase activity is greatly reduced in these cell types, so the role of WRN for maintaining genomic integrity and stability appears critical. We foresee the prospect of utilizing corrected WS iPSC to generate transplantable adult stem cells such as MSC for therapeutic purpose. Such approach holds two advantages. One is the global erasure of aging epigenetic marks and telomere reprogramming during integration-free and safe iPSC generation. The other one is the correction of disease-causing gene in pluripotent state rather than in more differentiated cells because clonal selection and expansion require substantial cell proliferation, which is telomere-sensitive.

## Recent advances in anti-aging research

**Fig. 2:**
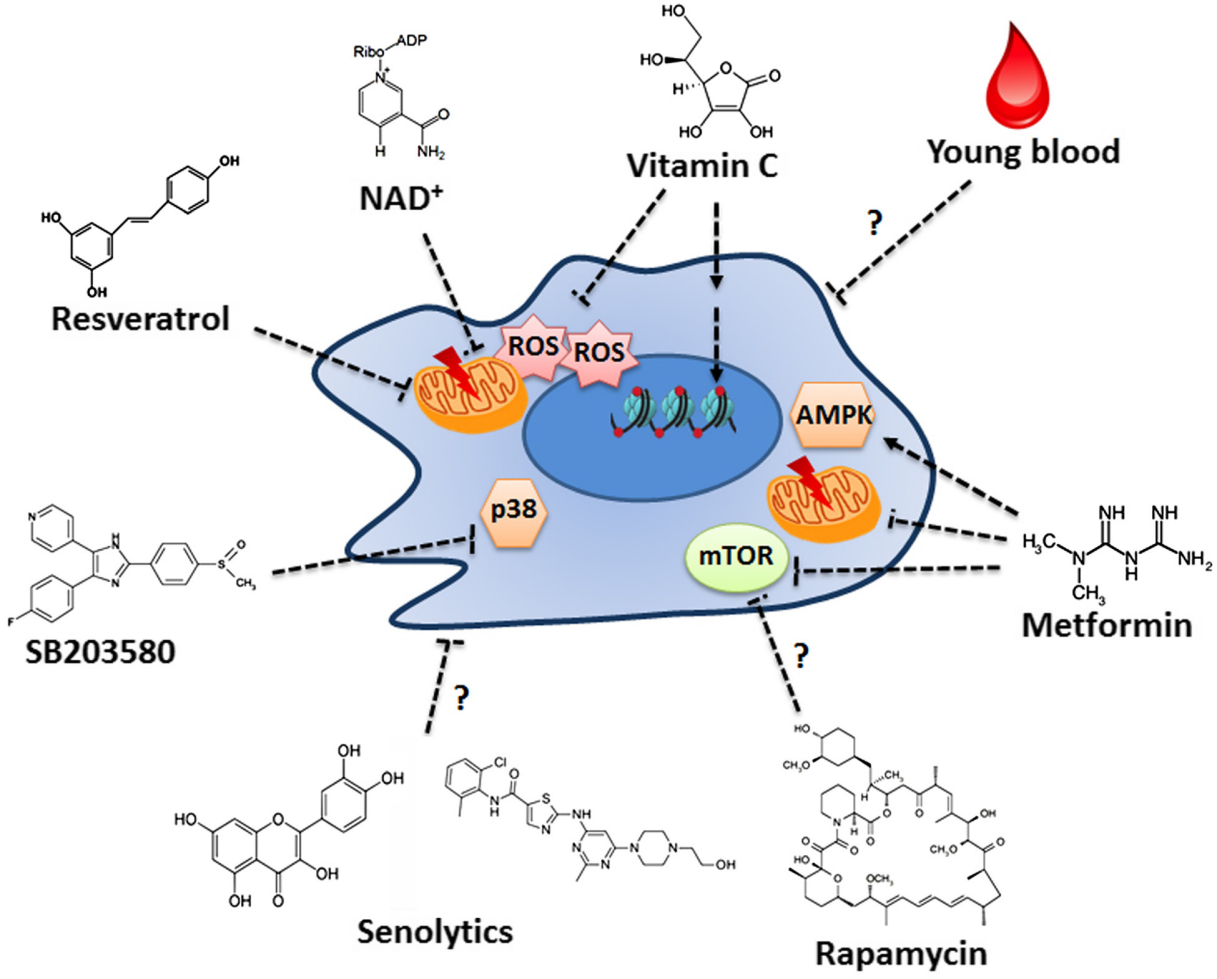
Drugs that improve aging cells. Resveratrol and NAD+ are known to improve mitochondrial dysfunction associated with aging, whereas vitamin C is an anti-oxidant for scavenging ROS as well as its additional role in epigenetic control. SB203580 is a p38 inhibitor that prevents premature senescence. Other compounds that have been shown to improve healthy aging may also benefit progeroid cells. Senolytics are drugs that selectively remove senescent cells, whereas rapamycin and metformin increase lifespan by targeting the mTOR pathway. Unknown factors from young blood may also improve aging in various organs

Progeroid syndromes share many similarities to natural aging, despite the different causes and origins driving the aging process. Reversal of aging through epigenetic reprogramming is still technically challenging. Anti-aging research on natural aging may also help developing drugs for treating premature aging diseases (Fig. 2). For example, vitamin C is generally used as an anti-oxidant for anti-aging. Moreover, it is shown to change the aging signaling pathway and improve healthy aging in the liver of a mouse model for WS [71]. Interestingly, the role of vitamin C is more than merely an anti-oxidant. It is surprisingly found to boost reprogramming of somatic cells to iPSC by alleviating senescence [72]. Vitamin C is later known to reduce the epigenetic barrier through regulating histone demethylases Jhdm1a/1b and DNA demethylation-related enzyme Tet1; both classes are critical epigenetic enzymes for reprogramming and senescence [73, 74]. The beneficial effect of vitamin C thus is not only limited to its anti-oxidation activity but also its ability to reset the aging-associated epigenetic marks. Adult WS patients mostly develop type 2 diabetes mellitus. Antidiabetic drugs like metformin, biguanide, and thiazolidinediones have been reported to improve insulin sensitivity in WS patients [75–77]. In addition to improve diabetes, metformin plays a second role in extending lifespan, as shown in animal models and clinical trials [78, 79]. Resveratrol, a natural product for anti-aging research, is also shown to improve insulin-resistant hyperglycemia and fatty liver in *Wrn* mutant mice, although with undesired side effects such as increased frequency of lymphoma and solid tumors [80]. Small molecules such as the SB203580 compound that inhibits the p38 MAPK signaling can ameliorate premature senescence in WS fibroblasts [81]. Such inhibitors are still in clinical trials. A number of anti-aging approaches have been recently discovered to improve healthy aging. These approaches, though they have not been experimentally and clinically tested for WS, may have therapeutic benefit on progeroid syndromes. For instance, age-related mitochondrial dysfunction (another hallmark of aging) is coupled with reduced nuclear NAD^+^ level, the process of which is dependent on SIRT1. Interestingly, increasing the NAD^+^ level in old mice restores mitochondrial function to a youthful state, raising the opportunity to reverse aging by modulating the mitochondrial function [82]. Senescence, an irreversible cell cycle arrest, is a driving force for organismal aging. Accumulation of senescence in an adult stem cell compartment is harmful to normal stem cells for their functions in differentiation, re-entering quiescence, and self-renewal. Inflammatory cytokines secreted from senescent cells also have a systemic effect on the pro-aging process [83]. Hence, removal of senescent cells is beneficial and has been demonstrated in model animals [84]. Drugs that selectively induce death of senescent cells may serve as an appealing alternative approach. A combination of drugs (dasatinib and quercetin) collectively termed senolytics is able to eliminate senescent cells in chronologically aged and progeroid *Ercc1* ^*−*/Δ^ mice, thus improving cardiac function and delaying the pathogenic symptoms [85]. It is exciting to see how such compounds can be translated into clinical uses and evaluation of safety. Recently, rejuvenation through the exchange of “youthful” factors from young blood is being underscored for the significance [86]. It was firstly demonstrated in a parabiotic pairing of young and old mice that share their circulatory system. This heterochronic parabiosis system improves stem cell function in multiple organs including the muscle, liver, heart, spinal cord, and even brain. Components from young blood help reverse age-related decline in skeletal muscle stem cells, cardiac hypertrophy, and improve cognitive function possibly through enhanced neurogenesis [87–92]. It remains largely unknown what “rejuvenation” factor(s) is responsible for such beneficial effect. Nevertheless, clinical trials using young blood have been approved for testing in people with Alzheimer’s disease and perhaps for progeroid syndromes in the future.

## Concluding remarks

Understanding the molecular mechanism driving adult stem cell aging will help us design drugs for combating aging. Progeroid syndromes are good models since the disrupted genetic pathways related to aging have been well studied. It is worth noting that biological aging is a consequence of multiple factors, each with its importance. This review only highlights the importance of telomere function and epigenetic regulation in WS because of the nature of the WRN helicase involved in these pathways. Other pathways such as ROS production, protein homeostasis, and mitochondrial dysfunction are equally important. Moreover, the aging mechanism of stem cells in different disease models does not completely overlap. The molecular mechanism leading to HGPS, for instance, is not identical to WS, although many similarities are found (e.g., epigenetic changes and accelerated senescence in mesenchymal cells). A more detailed comparison between different segmental progeroid syndromes can be found in other reviews [9, 93, 94]. To conclude, recent breakthroughs in studying age-associated diseases open new avenues to improve human health by targeting the aging pathways.
